# Correlation between Oxidative Stress and Thyroid Function in Patients with Nephrotic Syndrome

**DOI:** 10.4061/2011/256420

**Published:** 2011-10-13

**Authors:** Sangita U. Sawant, Subhash Chandran, Alan F. Almeida, M. G. R. Rajan

**Affiliations:** ^1^Radiation Medicine Centre, Bio-Medical Group, Bhabha Atomic Research Centre (BARC), Tata Memorial Hospital Annexe Building, Parel, Mumbai 400 012, India; ^2^Artificial Kidney Division, Department of Nephrology, King Edward VII Memorial Hospital, Parel, Mumbai 400 012, India

## Abstract

*Background*. The present study is to look for a correlation between oxidative stress and thyroid function in patients with the nephrotic syndrome in the remission phase as well as in a persistent proteinuric state. *Introduction*. Nephrotic syndrome is a form of chronic kidney disease due to which blood loses protein through the urine. We wanted to know if there was an increased loss of thyroid hormones in urine affecting thyroid function. *Methods*. 60 patients with nephrotic syndrome and 20 healthy non-proteinuric individuals as control subjects were enrolled in the study. We measured their serum tri-iodothyronine, thyroxine and thyroid-stimulating hormone. Estimation of lipid peroxidation (LPx) catalase, superoxide dismutase (SOD), and Glutathione peroxidase (GPx) were carried out by standard methods. *Results*. TSH was elevated in the nephrotic patients compared to controls, while TT4 and TT3 were significantly lower in the patients than in controls. Lipid Peroxidation and GPx were significantly higher in the nephrotic syndrome patients than in the controls, while SOD and catalase were significantly lower than in patients than in the control subjects. *Conclusion*. Nephrotic patients can lose significant amounts of thyroid hormones along with protein in urine, which can affect thyroid status, but this is reversible on remission.

## 1. Introduction

Nephrotic syndrome results in loss of plasma proteins and various other macromolecules in the urine leading to their deficiencies. Many of the physiologically important molecules which exist in the plasma, bound to plasma proteins, are also carried away and are lost in the urine [1]. The common abnormalities arising as a result of heavy proteinuria include hypothyroidism, vitamin D deficiency, and iron deficiency. While urinary loss of vitamin-D-binding globulin and transferrin accounts for their respective deficiencies, the exact cause of hypothyroidism in nephrotic syndrome appears to be complex. Initially it was thought that these individuals are metabolically euthyroid, as evidenced by their normal plasma free T4 (FT4) levels and free T3 (FT3) levels. The decrease in total T4 (TT4) was often attributed to the urinary loss of Thyroid Binding Globulin (TBG) and the resulting increase in unbound hormone suppressing further thyroid hormone secretion from the gland. As the thyroid gland function is regulated by thyroid-stimulating hormone (TSH) secreted from the pituitary gland, the levels of this hormone are expected to be low in the above case. However TSH levels were found to be very high in nephrotic syndrome and the increase in TSH correlates well with the degree of proteinuria. This leads to the possibility that significant amounts of thyroid hormones are also lost in proteinuric states resulting in a total body negative balance. In situations of long standing heavy proteinuria, it results in clinically significant hypothyroidism.

Whether the hypothyroidism of nephrotic syndrome remains an innocent bystander or plays an active part in the further course of the disease is yet to be understood. In most glomerular diseases, the renal injury is largely initiated by immunological mechanisms. However, once the injury is inflicted, subsequent progression to glomerulosclerosis and renal failure is mediated by nonimmunological factors like intraglomerular and systemic hypertension, tubuloglomerular feedback, glomerular hypertrophy and hyperfiltration, proteinuria, hyperlipidemia, and presence of extra renal disease. In recent years, researchers have been looking at reactive oxygen species (ROS) or oxidative stress as mediators of progressive glomerular injury [2–4]. 

Renal sources of ROS are activated macrophages, vascular cells, and various glomerular cells. ROS may affect cells of the host organism, especially at sites of inflammation, in addition to playing a role in the defence system against other agents. The effect plays a role in a variety of renal diseases such as glomerulonephritis and tubulointerstitial nephritis, which can contribute to porteinuria and other conditions. ROS are also thought to contribute to the pathogenesis of ischemia reperfusion injury in the kidney [5, 6].

Thyroid hormones (THs) are essential for normal metabolic function of the kidneys. Conversely, the kidney is not only an organ for metabolism and elimination of TH but also a target organ of some of the iodothyronines' actions. Thyroid dysfunction causes remarkable changes in glomerular and tubular functions and electrolyte and water homeostasis. Hypothyroidism is accompanied by a decrease in glomerular filtration, hyponatremia, and an alteration of the ability for water excretion [7–9]. 

Hypertension, diabetes, and proteinuria are well-recognized risk factors for progressive kidney function loss. However, despite excellent antihypertensive and antidiabetic drug therapies, which also often lower urinary protein excretion, there remains a significant reservoir of patients with chronic kidney disease who are at high risk for progression to end-stage kidney disease [10].

**Figure 1:**
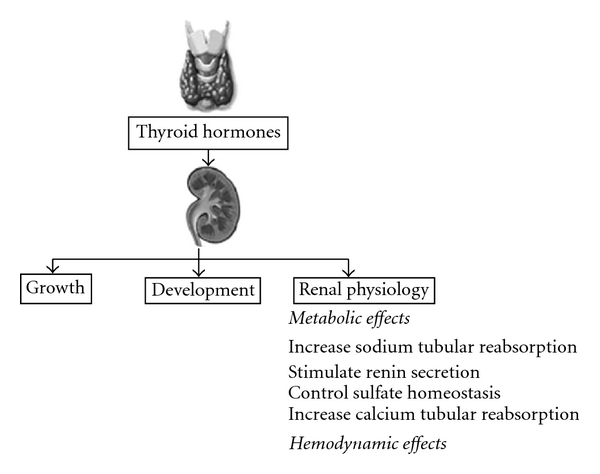
Effects of thyroid hormones on the kidney [11].

In order to ascertain whether the hypothyroidism of nephrotic syndrome has any role to play in glomerulosclerosis, we decided to estimate the thyroid hormone levels and markers of oxidative stress in patients with nephrotic syndrome, those in complete or partial remission and in healthy control subjects. We also tried to evaluate the correlation between oxidative stress and thyroid function in patients with the nephrotic syndrome in the remission phase as well as in a persistent proteinuric state (Figure 1).

## 2. Materials

The present study was carried out in the Radiation Medicine Centre, Bhabha Atomic Research Centre (BARC), Mumbai, in collaboration with the Department of Nephrology, King Edward VII Memorial Hospital (KEM), Parel, Mumbai. Sufficient and necessary Hospital Ethics Committee approvals from both institutions were taken.

A total of 80 subjects were enrolled in the study of whom 60 patients included in the patients were either in complete remission or had persistent proteinuria at the time of the study and 20 normotensive, nonproteinuric, healthy individuals formed the control group. 

The inclusion criteria were (A) Age between 12 and 50 years, (B) Documented nephrotic syndrome (defined as proteinuria of more than 3.5 gm/24 hr/1.73 m^2^ and (C) body surface area or any proteinuria associated with significant hypoalbuminemia, defined as serum albumin less than 2.5 gm/dL) at the time of enrolment to the study.

Regrouping of the 80 subjects, based on their 24-hour urinary protein excretion, the subjects were divided into four groups, control (0.15 gms/24 h), remission (0.09 ± 0.03 gms/24 h), subnephrotic (1.22 ± 0.90 gms/24 h) and nephrotic (5.07 ± 1.36 gms/24 h).

Both the patient and the control groups underwent a detailed study of history recording followed by complete physical examination with the purpose of ruling out secondary causes of glomerulonephritis and evidence of any systemic illness or infective focus. Routine laboratory investigations were done at K.E.M. Hospital.

Nephrotic syndrome occurring as a part of systemic disease such as diabetes mellitus, active infection, sepsis, smoking, alcoholism, and drug abuse and patients who had received steroids or immunosuppressant drugs within the 6-month period before enrolment were included the study.

## 3. Methods

We studied thyroid function and estimated (a) tri-iodothyronine (T3), (b) thyroxine (T4) by radioimmunoassay (RIA), and (c) thyroid-stimulating hormone (TSH) by immunoradiometric assay (IRMA) using in-house methods for T3 and T4 assays and BRIT kits for TSH. 

Estimation of lipid peroxidation (LPx) and antioxidant enzymes (superoxide dismutase (SOD), glutathione peroxidase (GPx), and catalase) in serum samples was as follows.

- Malondialdehyde content, the end product of the free radicals initiating lipid peroxidation, was measured by using thiobarbituric acid reactivity as described by Uchiyama and Mihara [12].
- SOD was measured by the method of S. Marklund and G. Marklund [13] as modified by Nandi and Chatterjee [14]. Superoxide anion radical is involved in the autooxidation of alkaline pyrogallol.
- The catalase activity was estimated by the method of Aebi [15]. Catalase degrades hydrogen peroxide, which can be determined by the decrease in absorbance at 240 nm in a spectrophotometer.
- GPx activity was analyzed by the method of Hafeman et al., based on the consumption of GSH [16]. Glutathione peroxidase degrades tertiary butyl hydroperoxide in the presence of GSH.

## 4. Results

### 4.1. Parameters of Oxidative Stress

**Figure 2:**
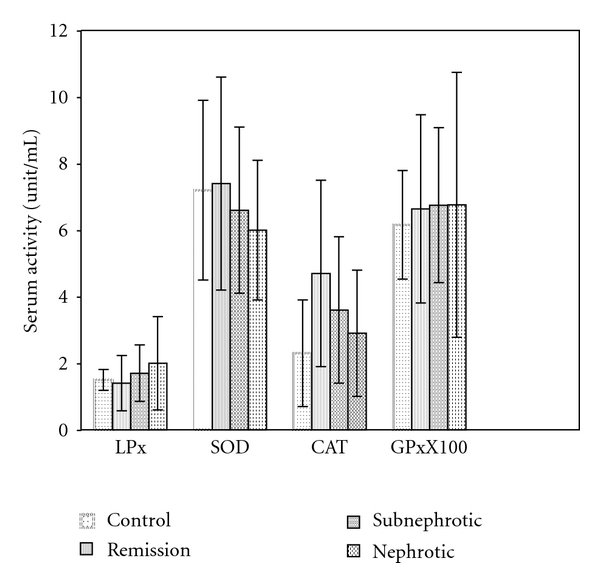
Parameters of oxidative stress in control, remission, sub-nephrotic, and nephrotic Groups.

All the parameters of antioxidant enzymes and lipid peroxidation are summarized in Table 1, and bar graphs in Figure 2 depict the parameters of oxidative stress in nephrotic syndrome.

The serum LPX levels the were lowest in the remission and subnephrotic groups with significantly maximum value in the nephrotic group.

Serum GPx was significantly higher in all the three groups in comparison to control group.

The serum SOD levels were significantly lower in the subnephrotic and nephrotic groups in comparison to control group, and also Catalase levels were significantly higher in all three groups in comparison to control with the minimum value in the nephrotic group.

### 4.2. Parameters of Thyroid Function Test

The estimates of TT3, TT4, and TSH are summarized in Table 2. 

As can be seen in Table 2, TT3 and TT4 levels were significantly lower and TSH was significantly higher in nephrotic syndrome than control group.

### 4.3. Statistically Analysis

All results are expressed as the mean ± SD. The data between control and test groups was compared using unpaired Student's *t*-test. Correlation was determined by Pearson's correlation coefficient. The level of significance used was *P* value less than 0.001.

### 4.4. The Results

The balance of reduction and oxidation (redox) of free radicals in the body is important to maintain health.

The Data suggest that the endogenous antioxidant enzymes serve as a mechanism of self-defense to prevent glomerular injury.

Superoxide dismutase enzyme is involved in catalyzing superoxide anion-free radical to produce hydrogen peroxide and singlet oxygen.

We have observed the decrease in SOD activity in nephrotic syndrome patients because superoxide anion produced during normal metabolic process could not be completely scavenged by the SOD. It is quite possible that it may accumulate and cause the initiation and propagation of lipid peroxides.

A decreased production of H_2_O_2_ would result in increased activity of catalase enzymes since hydrogen peroxide is hydrolyzed by catalase.

Both superoxide free radical and hydroperoxides can be generated within cells and could cause irreversiblility in activation of GSH peroxidase. GSH peroxidase functions to metabolize H_2_O_2_ created in the dismutation of superoxide radical and protects cell membranes for lipid peroxidation.

The serum levels of thyroid hormones were significantly lower in nephrotic patients compared to control, while the serum TSH levels were significantly higher as expected due to pituitary's response to the decreased hormone levels.

## 5. Discussion

Nephrotic syndrome is accompanied by changes in the concentrations of TH due primarily to loss of protein in the urine. Acute kidney injury and chronic kidney disease are accompanied by notable effects on the hypothalamus-pituitary-thyroid axis. Finally, data from a recent research suggest that TH, especially T_3_, can be considered as a marker for survival in patients with kidney disease [17].

Nephrotic syndrome is associated with an overall increase in oxidative stress. There is a concomitant statistically significant decrease in circulation of T4 and T3 levels and increase in serum TSH. The nature and severity of the original insult may possibly play role in contributing to the oxidative stress associated with nephrotic syndrome. Other than the decrease in thyroid function due to nephrotic syndrome (a form of nonthyroid illness), loss of thyroid-hormone-binding proteins in urine could be another cause of the decrease in serum T3 and T4 levels.

Bulucu et al. have studied the oxidative stress status in adult nephrotic syndrome by determining plasma selenium levels, erythrocyte and plasma GPx activities, erythrocyte Cu-Zn SOD, and erythrocyte and plasma levels of malondialdehyde (MDA). The 20 nephrotic syndrome patients had lower activities of SOD compared to controls, while erythrocyte and plasma levels of MDA were higher in the nephrotic syndrome groups [18].

Zachwieja et al.studied the total antioxidant status and mean antioxidant activity in 82 children with nephrotic syndrome of 4–16 years. The study suggested that reduced antioxidant activity in nephrotic syndrome may be related to lipid abnormalities [19].

Fydryk et al. examined the antioxidant status of 18 children with steroid sensitive nephrotic syndrome. In their study, the authors concluded that the majority of abnormal findings can be attributed to the hyper lipidemia of nephrotic syndreme (NS). Low GPx activity may be a factor limiting the antioxdiant capacity in NS [10]. 

Another study estimated the antioxidant status and reliable factor involved in antioxidant protection in children with nephrotic syndrome. The study suggested an increase in lipid peroxidation and insufficient antioxidant defense in Nephrotic syndrome [20].

The author concluded that, during remission, there is a tendency for the GSH-GSSG redox system to normalize [21].

Gilles et al. reported that abnormalities in thyroid function are seen in patients with proteinuria. Specifically, TSH levels were higher in patients with proteinuric renal diseases when compared with controls. Subclinical hypothyroidism occurred more frequently in the nephritic syndrome patients [22]. 

Adlkofer et al. examined the thyroid function of 13 patients with proteinuria and normal serum creatinine level (Group 1) and 15 patients with proteinuria and increased creatinine level (Group 2). The daily urinary T4 and T3 excretion was much higher in Group 1 patients than in Group 2 patients (37.1 ± 25.9 nmol versus 17.5 ± 8.7 nmol for T4 and 3.3 ± 1.6 nmol versus 1.1 ± 0.8 nmol for T3, resp.) and correlated in both groups with the protein loss [23].

Feinstein et al. showed that the reduced serum levels of TT4 and TT3 in these patients may be due to decreased binding to and/or concentration of serum carrier proteins, and, as in acute and chronic renal failure, patients with nephrotic syndrome with normal renal function have normal total and increased free rT3 values in association with reduced TT3 levels [24].

Hypothyroidism is also known to be accompanied by a decrease in glomerular filtration, hyponatremia, and an alteration of the ability for water excretion, which may indirectly reduce loss of protein through urine [7–9].

## 6. Conclusion

In hypothyroidism, thyroid hormone levels are very low, which suggests the possible direct involvement of free radical scavengers and lipid peroxidation. Increased glutathione peroxidase activity could be a compensatory mechanism in response to increased oxidative stress [25, 26].

Our finding suggests that nephrotic syndrome patients have an increased risk of subclinical hypothyroidism. Thyroid hormones accelerate the basal metabolic rate and more especially oxidative metabolism in vertebrates. Excess thyroid hormones may induce tissue injury secondary to production of active oxygen species. Thyroid function returns to the normal when the nonthyroid illness is resolved [27].

Proteinuria results in loss of thyroid hormones, most probably caused by loss of thyroxine-binding globulin along with T4 bound to it, thus stimulating TSH production [4].

Our data suggests that nephrotic syndrome patients may benefit from antioxidant therapy along with thyroid hormone supplement. 

## Figures and Tables

**Table 1:** Parameters of oxidative stress in nephrotic syndrome.

Group	Lipid Peroxidation nm MDA/mL	Superoxide Dismutase (SOD) U/mL	Catalase U/mL	Glutathione Peroxidase (GPx) U/mL
Control (*N* = 20)	1.5 ± 0.31	7.2 ± 2.7	2.3 ± 1.6	616 ± 163
Remission (*N* = 20)	1.4 ± 0.83	7.4 ± 3.2	4.7 ± 2.8*	664 ± 283*
Subnephrotic (*N* = 20)	1.7 ± 0.85*	6.6 ± 2.5*	3.6 ± 2.2*	675 ± 233*
Nephrotic (*N* = 20)	2.0 ± 1.4*	6.0 ± 2.1*	2.9 ± 1.9*	676 ± 398*

*P < 0.001.

**Table 2:** Urinary protein and thyroid hormone levels in nephrotic syndrome.

Group	Proteinuria (g/24 hrs.)	TT4 (*μ*g/dL)	TT3 (ng/dL)	TSH (*μ*IU/mL)
Control (*N* = 20)	<150	9.9 ± 2.6	130 ± 36	2.9 ± 1.2
Remission (*N* = 20)	111 ± 34	8.1 ± 2.9	132 ± 35	2.8 ± 1.3
Subnephrotic (*N* = 20)	1242 ± 898*	8.4 ± 1.5	119 ± 20	2.7 ± 1.0
Nephrotic (*N* = 20)	5137 ± 1282*	5.8 ± 1.6*	55 ± 30*	5.9 ± 1.8*

*P < 0.001.
